# International palliative care research priorities: A systematic review

**DOI:** 10.1186/s12904-020-0520-8

**Published:** 2020-02-03

**Authors:** Felicity Hasson, Emma Nicholson, Deborah Muldrew, Olufikayo Bamidele, Sheila Payne, Sonja McIlfatrick

**Affiliations:** 1 Institute of Nursing and Health Research, School of Nursing, Ulster University, Shore Road, Newtownabbey, BT37 0QB Northern Ireland; 2 UCD School of Nursing, Midwifery and Health Systems, UCD College of Health and Agricultural Sciences, University College Dublin, Belfield, Dublin 4, Ireland; 3 Academcy of Primary Care, Hull York Medical School, Allam Medical Building, University of Hull, Hull, HU6 7RZ England; 4 International Observatory on End of Life Care, Lancaster University, LA14YX, Lancaster, UK

**Keywords:** Palliative care, research priorities, Consensus, International. Systematic review

## Abstract

**Background:**

There has been increasing evidence and debate on palliative care research priorities and the international research agenda. To date, however, there is a lack of synthesis of this evidence, examining commonalities, differences, and gaps. To identify and synthesize literature on international palliative care research priorities originating from Western countries mapped to a quality assessment framework.

**Methods:**

A systematic review of several academic and grey databases were searched from January 2008–June 2019 for studies eliciting research priorities in palliative care in English. Two researchers independently reviewed, critically appraised, and conducted data extraction and synthesis.

**Results:**

The search yielded 10,235 articles (academic databases, *n* = 4108; grey literature, *n* = 6127), of which ten were included for appraisal and review. Priority areas were identified: service models; continuity of care; training and education; inequality; communication; living well and independently; and recognising family/carer needs and the importance of families. Methodological approaches and process of reporting varied. There was little representation of patient and caregiver driven agendas. The priorities were mapped to the Donabedian framework for assessing quality reflecting structure, process and outcomes and key priority areas.

**Conclusions:**

Limited evidence exists pertaining to research priorities across palliative care. Whilst a broad range of topics were elicited, approaches and samples varied questioning the credibility of findings. The voice of the care provider dominated, calling for more inclusive means to capture the patient and family voice. The findings of this study may serve as a template to understand the commonalities of research, identify gaps, and extend the palliative care research agenda.

## Background

Globally 40 million people are estimated to need palliative care each year, yet it has been estimated that only 14% are in receipt of such care [1]. Worldwide reports forecast that demand for palliative care is set to escalate over the next several decades, in response to changing demographics, longer disease trajectories and greater co-morbidity [2, 3]. Although palliative care has been advocated in global policy [4, 5] and viewed as a basic human right [6, 7], the proportion of research funding allocated is historically small [8, 9], resulting in a considerably under-developed evidence base [8]. For example, in the UK in 2013 the National Cancer Research Institute allocated 0.61% of its research budget to palliative and end of life care [10].

Priority setting is recognised as an essential task to help direct finite resources to support research [2, 11]. Such exercises are considered integral to the research process to guide and stimulate funding, fuel debate, and to strengthen the role of stakeholders in establishing the research agenda [12]. Ultimately, such approaches should help to underpin the development and improvement of palliative care for the patient and caregiver. In palliative care, a range of priority setting processes have been undertaken nationally across different countries and internationally by various organisations, networks and individuals [13–15]. A large proportion have been developed specific to disease type, for example, in head and neck cancer [16], dementia [17], intellectual disability [18] and generic palliative care [19] to name but a few. Priorities have also been identified by care setting [20, 21], patient demographic characteristics [22, 23], discipline focus [24, 25], and according to the specific components of palliative care such as pain [26] and spiritual care [27].

While it is important that the palliative care needs of specific disease groups and populations are considered and addressed, to enable transferable learning establishing global research priorities can provide a coherent research agenda, highlighting complex *multi-faceted global problems* [28, 29]. Moreover, such exercises can provide a platform for multidisciplinary research aiding the alignment of scarce global resources, so that important directions for future research can be met [30]. However, whilst global priorities provide a platform from which to understand the commonalities of palliative care, they underscore the complexity and unique characteristics of the landscape within which palliative care operates. For example, Zaman et al. [31] highlights the challenges of transferring ideals of palliative care between developed and developing countries, instead arguing for the need for global common denominators to be identified to enable culturally appropriate provision to be established.

Moreover mismatches between patient and health professional priorities have been reported [32]. This has spurred greater efforts to include patients and caregivers in the palliative care research agenda to ensure relevancy to their needs [33, 34]. The need to increase the value of resources invested in research is critical, and research that does not address the needs and concerns of its end users may be considered wasted [35] .

A preliminary search of the literature for previous systematic reviews of palliative care research priorities yielded no results. The need to establish the progress, and inform the development, of an international coordinated approach of palliative care research priorities is required to enhance transparency, identify and prioritise research topics, and ensure patients and caregivers are at the centre of that agenda. Therefore, this study aimed to identify and synthesize literature on international palliative care research priorities, originating mainly from Western countries, mapped to the Donabedian framework [36]. Critically, the review also synthesised the approaches adopted, stakeholders involved and the jurisdictions in which the priorities have been developed. Using thematic synthesis [37], a set of high-level research priorities have been developed to provide the basis for a strategic international framework for palliative care research going forward.

## Methods

### Study design

A systematic review of research priorities in palliative care was undertaken and guided by the PRISMA statement for reporting systematic reviews [38] .

### Search strategy

A systematic search of databases from health sciences, medicine, and psychology was undertaken in August 2017 in conjunction with a subject librarian. Six databases were searched: Cumulative Index of Nursing and Allied Health Literature (CINAHL), Excerpta Medica database (EMBASE), PubMed, SCOPUS, Web of Science, and PsycINFO. Grey literature was identified via ProQuest Dissertations and Theses, CareSearch grey literature, James Lind Alliance Website, Lenus, and the Palliative Hub of the All Ireland Institute of Hospice and Palliative Care. A further search of the grey literature was conducted on the following sites in April and May 2019: OpenGrey, European Association of Palliative Care (EAPC) conferences, Australian and New Zealand Society of Palliative Medicine and Google.

Key words were identified through the titles, abstracts, and indexed phrases of relevant articles from a preliminary search of PubMed and CINAHL. Indexed terms from the selected databases were identified and included in the search terms for these specific databases (Table 1). Articles were limited by publication date (January 2008–June 2019) and language (English). Subsequently, indexed terms (e.g., CINAHL headings, mesh terms) from the selected databases were identified and included in the search terms for these databases

**Table 1:** Search terms used in the systematic review (including an example of the Mesh terms from the search of PubMed)

“Palliative care” OR “end of life” OR “terminal care” OR “Critical care” OR hospice OR “terminally ill” OR “Palliative Care”[Majr] OR “Terminal Care”[Majr:NoExp] OR “Hospice Care”[Majr] OR “Terminally Ill”[Mesh] AND	
“Research priorit*” OR “Health services research” OR “Research Agenda” OR “Research quest*” OR “Research Gap*” OR “Knowledge gap*” OR “Research initiative*” OR “Research recommendation*” OR “priority areas of research” OR “Evidence Base” OR “Research Subject*” OR “Policy-relevant research” OR “Research program*” OR “Research direction*” OR “Recommendations for research” OR “High-quality research” OR “Research”[Majr:NoExp] OR “Health Services Research”[Mesh:NoExp]	

### Screening

**Fig. 1:**
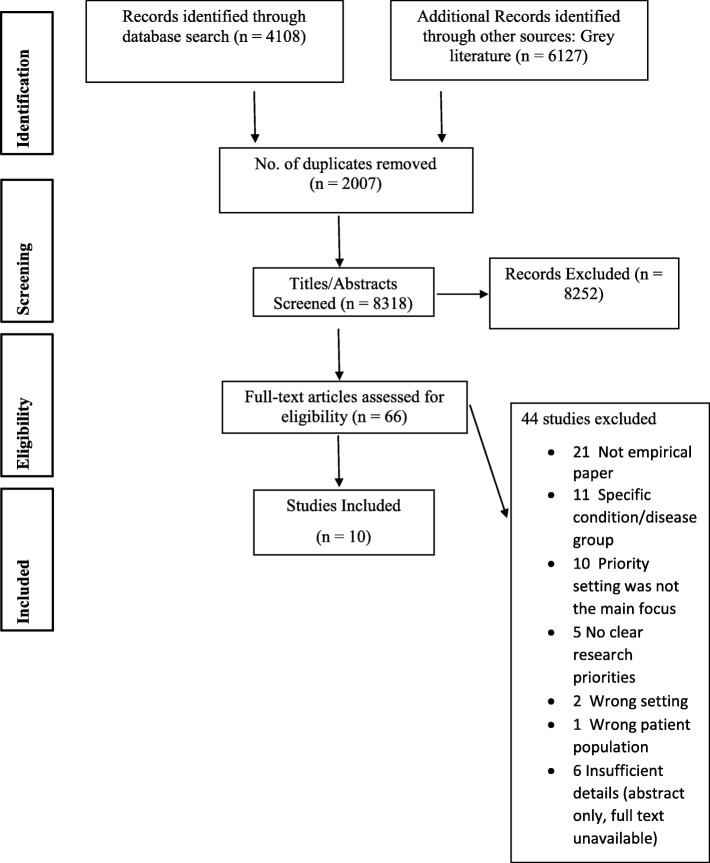
PRISMA Flow Diagram

The search yielded 10,235 articles from which 2007 duplicates were removed (Fig. 1). Two reviewers (EN and FH) uploaded titles and abstracts of the remaining 8318 papers into Covidence for initial screening, of which 8252 were considered irrelevant and excluded. Four reviewers (EN, DM, FH and OB) screened the full texts of the remaining 66 articles against the review’s inclusion/exclusion criteria (Table 2).

**Table 2:** Inclusion and Exclusion criteria for the systematic review

Inclusion Criteria	Exclusion Criteria
Studies that directly elicited and identified research priorities for palliative care (including patients/carers, healthcare providers, policymakers, and researchers) and parentheses	Studies that have considered research priorities relating to specific aspect of palliative care (e.g., spiritual, medical)
Methods of identifying priorities could include (but not limited to) surveys, qualitative studies, consensus methods (Delphi survey, nominal group technique), and workshops	Studies assessing priorities for practice and policy (quality indicators); non-research articles (policy documents, clinical guidelines, editorials, commentaries); reports of a conference, workshop or meeting that did not include information about the participants and methods; and basic science research, epidemiological studies, guidelines, and economic evaluations were excluded.
Studies published in English	Excluded studies with an exclusive focus on populations with specific palliative care needs such as intellectual disability, paediatric, adolescent, or geriatric populations

Ten studies were eligible, and they were included for quality appraisal (see Fig. 1). Any conflict of opinion regarding which article to include or exclude was resolved through discussion among the reviewers. If no agreement could be reached between the reviewers, a fifth reviewer (SM) mediated.

### Quality appraisal and risk of Bias

Quality appraisal was undertaken to gain an understanding of the results and level of confidence in the findings. The quality, methodological rigour, and risk of bias of the ten studies included in the final review were assessed using the Joanna Briggs Institute (JBI) Critical Appraisal Tools [39]. JBI critical appraisal tools were selected based on their appropriateness to the methodology in the papers. The qualitative research tool [40] was utilised for papers using workshops, the systematic review tool [41] was used for evidence reviews, and the cross-sectional research tool [42] for research using surveys and questionnaires. To prevent the introduction of bias and enhance transparency, four studies [21, 43–45], adopting multiple methods and consensus methods, were not subject to quality appraisal due to a lack of specific tools. No study was excluded based on quality.

Quality assessment for included studies was completed independently by EN & DM. Any unresolved variances were resolved by a third author (FH). These appraisals were summarised respectively and presented the grading using a range of coding systems. The scores were computed by counting the number of `Yes’ answers. A coding system of 0–10 was applied to three papers; 8–10 was considered high quality, 4–7 moderate and below 4 poor. Of the three papers one was considered high and two moderate quality. Two papers were accessed using a coding system of 0–8: 7–8 was considered high quality, 6–4 moderate and 3–1 poor. The two papers were each accessed as moderate quality. The remaining paper was accessed using coding system of 1–11; 8–11 was considered high quality, 4–7 moderate and below 4 poor. In terms of quality this paper was rated as high quality (see Table 3).

**Table 3:** Quality Appraisal

	Q1	Q2	Q3	Q4	Q5	Q6	Q7	Q8	Q9	Q10	Q11	Grade
JBI Qualitative												
Diffin et al	Y	Y	Y	Y	Y	Y	N/A	Y	U	Y	–	8/10
Pillemer et al	N/A	Y	Y	Y	Y	U	N/A	Y	N/A/	Y	–	6/10
Powel et al.	Y	Y	Y	Y	Y	Y	N/A/	Y	N/A/	Y	–	8/10
JLA PSP	Y	Y	N/A	N/A	N/A	N/A	Y	Y	–	–	–	4/8
JBI Systematic review												
Riffin et al.	Y	Y	Y	Y	U	U	Y	Y	U	Y	Y	8/11
JBI Cross sectional												
Perkins et al	Y	Y	N/A	Y	U	U	U	Y	–	–	–	4/8
Key												
	JBI Qualitative Checklist				JBI Systematic review				JBI Cross sectional			
Q1	Is there congruity between the stated philosophical perspective and the research methodology?				Is the review question clearly and explicitly stated?				Were the criteria for inclusion in the sample clearly defined?			
Q2	Is there congruity between the research methodology and the research question or objectives?				Were the inclusion criteria appropriate for the review question?				Were the study subjects and the setting described in detail?			
Q3	Is there congruity between the research methodology and the methods used to collect data?				Was the search strategy appropriate?				Was the exposure measured in a valid and reliable way?			
Q4	Is there congruity between the research methodology and the representation and analysis of data?				Were the sources and resources used to search for studies adequate?				Were objective, standard criteria used for measurement of the condition?			
Q5	Is there congruity between the research methodology and the interpretation of results?				Were the criteria for appraising studies appropriate?				Were confounding factors identified?			
Q6	Is there a statement locating the researcher culturally or theoretically?				Was critical appraisal conducted by two or more reviewers independently?				Were strategies to deal with confounding factors stated?			
Q7	Is the influence of the researcher on the research, and vice- versa, addressed?				Were there methods to minimize errors in data extraction?				Were the outcomes measured in a valid and reliable way?			
Q8	Are participants, and their voices, adequately represented?				Were the methods used to combine studies appropriate?				Was appropriate statistical analysis used?			
Q9	Is the research ethical according to current criteria or, for recent studies, and is there evidence of ethical approval by an appropriate body?				Was the likelihood of publication bias assessed?							
Q10	Do the conclusions drawn in the research report flow from the analysis, or interpretation, of the data?				Were recommendations for policy and/or practice supported by the reported data?							
Q11					Were the specific directives for new research appropriate?							

### Data extraction

A data extraction form was developed on Microsoft Word to extract key data from the included studies. Data extracted included author, year, and aim of the study, geographical location, participants, method, data analysis and priorities identified. Three reviewers (EN, DM and OB) independently extracted data from the final ten papers using the data extraction form and any disagreements were resolved through discussion. A fourth reviewer (FH) was consulted if an agreement could not be reached.

### Data synthesis

Categorical data including year, country, participants, and method were extracted and analysed in Microsoft Excel. Qualitative data underwent a thematic synthesis [37] to integrate the findings of multiple studies and identify which priorities were the most common across the ten included papers and this allowed for the development of higher order themes. Synthesis included line by line coding of the findings of the primary studies, and the categorisation of codes into broad groups of research priorities followed by descriptive themes [37, 46]. The final stage in the analysis was the development of broad analytical themes.

## Results

### Overview

The data extracted from the ten included studies [15, 21, 43–50] are presented in Appendix. The largest group to shape the research priorities were academic, commissioners and healthcare professionals (HCPs) and these were included in eight of the ten studies [15, 21, 43–45, 47, 49, 50]. Families and carers were the second largest group to contribute to the data and were included in two studies [15, 47] . Patients were the sole contributor in one study [48] and contributed as part of a group of patients and families in two other studies [21, 43]. However, these were the only studies in which patients shaped the research priorities. Six of the studies included researchers [15, 43–45, 49, 50], two included researcher/clinicians [43, 50], one included members of the public [15] and two included palliative care volunteers [15, 43]. Details on the sample sizes for each participant group can be found in Appendix.

One study included a search of the international literature and as a result the data was not exclusive to one jurisdiction [46]. The geographical location of the studies was diverse. Three of studies were based in the United Kingdom [44, 47, 48], while one was conducted in both the United Kingdom and Ireland [20]. Each of the remaining five studies geographical location were conducted in New Zealand [21], United States [49], Canada [43], Australia [45] and Africa [50] respectively.

Consensus methodologies were the most commonly used method of developing research priorities. However, there was little consistency in how consensus was gained across the studies as methods were operationalised in different ways. For instance, two studies [21, 45] utilised the Delphi technique while others used workshops [15, 47, 49], a nominal group technique [44, 50] or a mixed methods approach involving literature review interviews and online surveys [43] respectively. One study used a systematic review methodology alongside an innovative analytical approach to synthesise evidence from review articles or consensus reports to develop a list of research priorities [46]. One study used a questionnaire as the only method for developing the priorities [48] while others incorporated surveys as one phase of a single priority-setting exercise [15].

### Descriptive themes and priority areas

Following the thematic synthesis [37], the data from the studies were organised into seven descriptive themes, which are described in more detail below.

#### Service Models

This theme focused centrally on the provision of out of hours (OOH) care and home care services across all disease groups [43, 44]. Understanding community care provision, resources, and models and barriers to 24-h care underpinned this theme [15, 21, 43–45, 47–49], with a particular focus on explicating the benefits of home care, understanding and meeting patients’ needs, and mechanisms to maintaining independence and enabling patients to remain at home. There is need for better understanding and implementation of a model of care which identifies and delivers the palliative care needs of non-cancer patients in the community [44] and other non-hospital settings (such as primary care or nursing homes) [49]. Researchers called for a systems-level approach to “*develop innovative models for delivering palliative care to community-dwelling patients*” [49]. Research evidence is also needed to facilitate the transformation of care from the existing medical model to a person-centred public health approach which uses an organised community effort to provide compassionate care and support for people with life-limiting conditions and their families (compassionate community model of care) [43]. More generally, the efficacy of different models in terms of outcomes and cost-effectiveness was also recognised as an important area for research [43].

#### Continuity of Care

This theme relates to research that recognises the interdisciplinary nature of palliative care delivery with a view to facilitating greater continuity across all services related to palliative care, to decrease the number of HCPs that patients come into contact with while in receipt of palliative care, as well as exploring how patients transition between services. Specific topics for research included examining the impact of a “*designated case coordinator”* [15] or exploring how to implement “*effective partnering with other providers and specialists in the care of palliative patients*” [21]. Improved communication between primary care and hospital was also recognised as a priority for future research that may support greater continuity of care.

#### Training and Education

The training and development of non-palliative care specialists was cited as a critical area for research with primary care providers as the main group of HCPs that should be targeted for further training in palliative care, as well as non-hospital based providers [15, 21, 43, 46, 47, 49, 50]. Research evidence is also needed to inform content and implementation of training programs for health care professionals on early integration of palliative care [43]. Specifically, future research needs to identify the training needs of primary care providers e.g.*, “investigate the support and education needs of general practices for provision of palliative care in primary health*” [21] and assess “*the impact of these programs on both provider practice and patient outcomes*” [49]. Additional training for hospice staff and palliative care specialists was also viewed as important with a focus on identifying critical areas for further training, exploring and improving practices regarding palliative care for dementia, and how to engage staff in further training. Moreover, testing and developing training and education programmes for non-professionals such as families and carers was also a priority.

#### Inequality of Access

This is a broad theme that incorporates issues pertaining to inequality of access to palliative care services due to diagnosis and a lack of knowledge around disease trajectories for “*patients with conditions other than cancer*” [49]. Moreover, the paucity of evidence around the cultural and social factors that influence access to palliative care was also highlighted in many of the studies with respect to a “*need for equal access to care across different diagnosis groups, socio-economic status and geographical location*” [47]. There is need for research to inform interventions to promote equitable access to quality palliative and end-of-life care tailored to meet patient’s unique needs especially among hard to reach [43], indigenous [45] and other marginalised groups (such as non-cancer patients) [44].

#### Communication

This theme encompasses all aspects of communication in palliative care; there is a need for evidence that will improve communication at every level (e.g. *“patient---family or patient--provider decision-making and communication”)* [43, 45, 46]. This includes communication between services, across specialities, between services and patients, services and families, and patients and their families/carers to facilitate their understanding of transition from active treatment to palliative care [15, 43, 45–49]. There is further need to investigate ways by which accurate information about patient’s prognosis can be best communicated to them and when [45]. For example, “*Helping doctors to hear and understand what patients are saying*” [48] as well as establishing “*better ways to make sure there is good communication between doctors working in different places”* [48] were cited as two key areas where communication can be improved.

#### Patient Preference and Experience

This theme incorporates priorities related to specific patient needs and outcomes around the treatment of symptoms (both physiological and psychological) that hinder their ability to live well and with autonomy. Patients are keen to be independent for as long as possible and little is known about their lived experiences of palliative care and “*the sense of loss for patients in not being able to participate in activities and hobbies they have previously enjoyed*” [21]. Future research should aim to find an appropriate balance between HCP involvement and patients’ needs and goals as well the “*management of both the patient and carers, and HCPs expectations in relation to their involvement in various aspects of care”* [47]. The theme also focuses on goal setting for individual patients so that palliative care outcomes are targeted to their own needs and that research should emphasize “*care outcomes and the impact of palliative care as perceived by patients*” (48)p39. Research is further needed to inform how the quality of care can be optimised by identifying better ways of managing patient’s pain and symptom and reduce the toxic impact of experimental cancer treatments [43].

#### Recognising the needs and importance of Family Carers

This theme outlines the necessity for research that provides a greater understanding of the “*needs of families, caregivers”* [15, 50] of palliative care [43, 44]. Given the holistic nature of palliative care, research should be wary of isolating patient experiences from those of families/carers and cognisant of the system of support provided to patients by families/carers and the knowledge of the patient they bring to research. Research to promote a better understanding of effective strategies to improve patient and families’ involvement in decision-making regarding end-of-life care for the patient and bereavement support for family, was further highlighted as a priority [45]. Finally, establishing “*the education/training support needs of carers*” [47] was highlighted as a key area of research for this population, in particular with regards to the care they provide at home, for example, “*find out what it is like for family member/caregivers to have responsibility for monitoring patient changes and adjusting medications in the home*” [21].

### Analytical themes

The seven themes that emerged from the thematic synthesis were closely aligned to the data extracted from the ten included studies. However, it was important to further interpret this data in order to generate a higher-order explanation for the findings. The Donabedian Framework [36] is a model of assessing quality of care and provides a useful mechanism for displaying and analysing this data. This framework will help to provide a standardised model to summarise the research priority results according to quality indicators and identify gaps in the evidence.

**Fig. 2:**
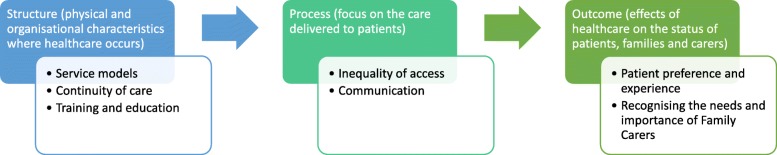
Analytical Themes incorporated into the Donabedian Framework (1966) for quality of care

The seven descriptive themes are reflective of and mapped onto the three interrelated categories contained within the Donabedian framework, where each category influences the one that succeeds it. Structure refers to the attributes of the settings in which palliative care is delivered. Process involves the activities, from both professionals and patients, that are carried out in giving and receiving care. Outcomes denote the impact of the care on patients and families [51]. Three of the themes (Service Models, Continuity of Care, Training and Education) are centred on structure and the physical and organisational features of palliative care service provision. Inequality of Access and Communication are elements of the process of care delivered to the patients While Patient Preference and Experience and Recognising the needs and importance of Family Carers are related to the outcomes of care for these populations (see Fig. 2).

### Contribution of the included studies to the final themes

The relative contributions of each of the ten papers to the final themes was examined and the data is displayed in Table 4. Each of the ten studies contributed at least three of descriptive themes and only one study [50] did not contribute to all three analytical themes (as outlined in the Donabedian Framework). Two of the studies [15, 49] contributed to all seven descriptive themes.

**Table 4:** Representation of the seven descriptive themes in the included studies mapped to the Donabedian framework

Donabedian framework		De Vries et al. 2016	Diffin et al. 2017	Pan- Canadian Framework 2017	PeoLPSP et al. 2015	Perkins et al. 2008	Pillemer et al. 2015	Powell et al. 2014	Riffin et al. 2015	Shipman et al. 2008	Sullivan et al. 2018
Structure	Service Models	X	X	X	X	X	X			X	X
	Continuity of Care	X	X		X		X		X		
	Training and Education	X	X	X	X		X	X	X		
Process	Inequality of Access		X	X	X		X		X	X	X
	Communication		X	X	X	X	X		X		X
Outcomes	Patient Preference and Experience	X		X	X	X	X	X	X		X
	Recognising the needs and importance of Family Carers	X		X	X		X	X		X	X

## Discussion

### Main findings of the review

Seven priority areas were identified from the ten papers included in the systematic review; Service Models, Continuity of Care; Training and Education; Inequality of Access; Communication; Patient Preference and Experience; and Recognising the needs and importance of Family Carers. The themes were mapped to the Donabedian Framework [36], which highlighted that the priorities were associated with the setting, structure variables and the effects on patient outcomes. Despite research emphasising the inclusion of patients and caregivers in research, only five studies [15, 21, 43, 44, 48] included the patient and/or caregiver. The need to place the patient at the centre of this process in line with policy is advocated in many countries. This review also analysed the methods used for priority setting, indicating varied approaches were adopted. The majority were based upon consensus methodology however, the operationalisation of consensus and transparency of the process was lacking. This confirms previous work by Viergever and colleagues [28] which suggests there is no gold standard for setting research priorities emphasising the need to improve the rigorous and transparent reporting of methods.

### What is already known and what does this review add

Internationally there is a paucity of research considering the priorities for palliative care research originating from Western countries; only ten papers were identified that contribute to priority setting relevant across palliative care provision. While a proportion of the literature in this area focuses on palliative care provision for individual disease-groups and conditions, it is important to recognise the opportunities for shared learning and commonalities across all populations. This review is the first to synthesize international research priorities for palliative care that have been obtained from empirical research and that are not disease or population specific, onto a tangible framework within the broader healthcare context. The Donabedian framework, has been successfully applied across healthcare settings and contexts as a means of evaluating quality, and within the palliative care context, for example, as a systematic review framework [52], and as a proposed framework for intervention and evaluation studies [53, 54]. The utilisation of this framework transformed the thematic synthesis into higher order analytical themes that can be taken forward strategically to improve palliative care research. To the authors’ knowledge, this is the first systematic review to apply the Donabedian’s framework to mapping palliative care research priorities.

Palliative care is a field of healthcare in receipt of a historically small proportion of research funding [8, 9], therefore, it is essential to ensure that research value is maximised. In 2009, Chalmers and Glasziou [55] estimated that 85% of all health research is being avoidably “wasted”. Whilst it can be argued that progress has been made, over the last decade, Glasziou and Chalmers more recently [56] claimed that health service research still has a have a long way to go, with continued concerns over research design, conduct and reporting. When analysing the priority areas identified, it is important to be cognisant of these concerns and ensure that the palliative care research projects to address these priorities are of sufficient quality and rigour to address and mediate such concerns.

One of the central issues about priority setting is addressing the question around who should be involved in the process, and how can this be enabled. Findings from this review suggests the care providers are the dominant perspective, with the patient’s largely missing. This may explain why priorities are largely service orientated with notable gaps in the priorities relating to quality of life and symptom management. Given that patient perspectives routinely differ from those of other stakeholders [57], the need to elevate their voice to enhance the legitimacy in the identification of priorities is required. Doing so will help to ensure that future research addresses questions of relevance, helping decision-makers and service providers to be better equipped to design and deliver health services to meet patient/service user need [58]. Whilst researchers have acknowledged the need for greater patient involvement in research and planning [59], their inclusion is questioned by an array of ethical, practical and medical challenges [60], further complicated by researcher concerns about their roles and values [61, 62]. Nevertheless, the need to find new robust processes to support meaningful contribution of patients to enable inclusion in strategic directions in palliative care is required to ensure the inequality [2] is reduced and the research is not considered wasteful [35].

A second issue relates to how information is collected. Variations of methodological approaches exist, with little agreement on what constitutes reporting standards, guidelines or best practice [28, 63]. Findings in this study demonstrate great variation in the approaches used to organise research priority-setting exercises. Whilst consensus was the most common means to generate research priorities in the ten included studies, a myriad of approaches were used such as the Delphi technique, nominal group and consensus workshops, as well as a range of analysis techniques, for instance, ranking, statistical analyses and an immersion crystallisation framework. Within the ten studies, inconsistent reporting of priority development was noted and was a barrier when synthesizing the evidence across studies. Such variation and lack of consistency make it difficult to judge the validity and transferability of the priorities reported, creating a significant barrier to aggregating and reporting comparative findings across international contexts [63]. Given that credibility of consensus findings is influenced by the rigour application of the approach, we need to ensure the reporting and guidance by acceptable standards in palliative care is advocated [2, 64]. Moreover the application of the checklist to help standardise research priority setting in health could be used to inform priority setting exercises going forward [28].

A third issue relates to the context and landscape in which the priority setting is undertaken. In this review, the majority of priority setting exercises were conducted in countries with an preliminary (i.e., New Zealand) or advanced (i.e., UK, Ireland, U.S.A, Canada and Australia) level of palliative care programmes and integration into mainstream health services [65]. A gap in the knowledge of priorities representing those in lower and middle-income countries (LMIC) exists, echoing previous reviews of international palliative care research [66, 67]. Whilst there is evidence in this review of engagement, with researchers from high-income regions collaborating and undertaking research in Africa (Powell et al.*,* 2014), a number of researchers [31, 67] suggest this raises multi-faceted challenges including the risk of imposing western norms in differing cultural contexts [68]. Therefore, the application of western research priority findings is limited, if not adapted to the specific economic, cultural and specific health care context and constraints of lower- and middle-income countries. Zaman et al. [31] suggests the need for LMIC to initially develop culturally and locally appropriate research, and then move towards international comparative research.

### Strengths and limitations

This review represents an initial step towards mapping international palliative care research which may help to inform policy and funding bodies on future action. However, it has several limitations for example, the search was limited to English language articles, which limits the generalizability of the findings. Moreover, it is recognised that this review excluded, disease specific empirical studies such as dementia and for specific populations such as intellectual disability however, the inclusion of such evidence would have resulted in greater heterogeneity between studies and limited the ability to synthesise the findings. The search was also limited that the exclusion of patient and public involvement which may have captured more caregiver and patient perspectives. Additionally, some of the studies lacked detailed information on the methodological analysis and procedures employed, questioning the rigour and validity.

### Implications for policy, practice, and research

This systematic review has called attention to the need for more end users in research priority setting exercises, therefore, researchers and funding bodies should develop new strategies to ensure meaningful participation of palliative care patients and families, building in structures and processes to account for the vulnerability often present within this population. Findings provide an initial blueprint for palliative care research funders and policymakers to contribute to the future research agenda for palliative care from a patient and HCP perspective. Given that funding resources are limited the importance of collaboration and international approaches to palliative care is growing, these findings may help to inform this debate.

Methodologically, a standardised approach and reporting for priority setting is advocated allowing for increased validity and comparability of findings from across palliative care settings. Due to varied methods and analytical techniques, an additional challenge was presented for the authors of this review when trying to compare and weight studies. Future attempts to set research priorities should involve a multi discipinary representation of stakeholders, such inclusion will provide credibility and enhance the feasibility of the developed priorities. Whilst it is outside the remit of this review to specific an appropriate prirotiy setting methodology, the conduct of any such exercises should be governed by methodological guidelines, clear objectives and defined criteria and concepts, for identifying and ranking priorities. Doing so, will aid the transparency of the process and credibility of the results.

## Conclusions

A review of the international palliative care priorities generated a list of common denominators within the palliative care landscape. However, it is unclear if they align with the needs and concerns of the patent and caregiver who are at the centre of palliative care. In addition, the reporting of the priority process was ambiguous which raises questions regarding the credibility of findings. The findings of this study may serve as a template to understand the commonalities of research and enhance dialogue in palliative care research.

## Data Availability

The datasets used and/or analysed during the current study are available from the corresponding author on reasonable request.

## Appendix

**Table 5:** Data extraction

Authors	Year	Aim of Study	Geographical Location	Participants	Methodology	Data Analysis	Priorities Identified (summary^a^)
De Vries et al.	2016	To inform organizational decision making and policy development regarding future research priorities for a hospice service in New Zealand	New Zealand	Palliative care staff (*n* = 10, 18, and 9 per round 1–3) volunteers (*n* = 10, 12, 11 per round) patients and family members (*n* = 6, 8 for round 1 and 2), and community linked professionals (*n* = 3)	Modified Delphi Technique	Descriptive statistics. For each question, the proportion of scores of four or more were calculated and ranked to identify the 48 most preferred topics	Patients and Families: • Decision-making • Bereavement and loss • Symptom management • Recognition of need and response of service Staff and Volunteers: • Symptom management • Aged care • Education • Community • Patient/family • Bereavement & support for young people
Diffin et al.	2017	The aim of this project was to identify EoL research priorities specific to Greater Manchester via a consultation process with both healthcare professionals (HCPs) and carers	Greater Manchester (United Kingdom of Great Britain and Northern Ireland)	Healthcare Professionals from Greater Manchester (*n* = 32)/ Family carers from greater Manchester (*n* = 26)	Initial Scoping followed by consultation through informal workshops and interviews	Data organised under six main topic areas and ranked	Top 3 priorities for both groups: 1. Access to 24 h care 2. Planning end-of-life care in advance 3. Staff and carer education Common themes: • Need for improved communication between stakeholders • Need for equal access to care • Management of both the patient and carers, and HCPs
Palliative and end of life care Priority Setting Partnership	2015	To identify unanswered questions which are most important for people in their last years of life, current and bereaved carers, and health and social care professionals	United Kingdom and Ireland	1403 initial survey participants (48% professional; 35% bereaved carers; 13% current carers; 10% other; 4% patients; 3% volunteers)^**a**^ 1331 interim prioritisation survey participants (64% professional; 22% bereaved family/carer; 9% current family /carers; 11% other; 8% public; 2% patient)^**a**^ 24 workshop participants ^a^overlap reported in categories as respondents reported as belonging to more than one category	James Lind Alliance Methodology Initial survey generated 83 Qs. Ranking of 83 priorities. Workshop (NGT) ranked top 28 questions to result in 10 priorities	Ranking	Top 10 in order of priority: 1. The best ways of providing palliative care outside of working hours. 2. Improving access to palliative care services be improved for everyone regardless of location? 3. Benefits of Advance Care Planning and other approaches. 4. Information and training for carers and families 5. Ensuring staff, including healthcare assistants, are adequately trained. 6. Determining palliative care needs for patients with non-cancer diseases 7. Core palliative care services regardless of diagnosis. 8. Benefits of providing care in the patient’s home 9. Ensuring continuity for patients at the end of life. 10. Assessing and treating pain and discomfort in people at the end of life with communication and/or cognitive difficulties.
Pan-Canadian Framework for Palliative and End –of-Life Care Research	2017	To develop a research framework for palliative and end-of-life care	Canada	36 Interviews with individuals drawn from a number of stakeholder groups (patients, caregivers, health care practitioners, health care administrators, opinion leaders and others with an interest in palliative and eol issues. 172 completed surveys (51 patients/caregivers; 41 practitioners; 62 Researcher/Clinician Researcher; 13 decision makers; 5 volunteers)	Literature review (2005–2013), interviews (face-to face and by telephone and online survey	Thematic grouping	Priority research areas identified under three broad themes and eight sub-themes: 1. Transforming model of care a. Engaging communities using a public health approach b. Early and integrated palliative care c. Access to quality palliative and end-of-life care 2. Patient and family centeredness a. Pain and symptom management b. Optimising quality of care c. Person-reported outcomes 3. Ensuring equity a. Addressing the needs of special populations b. Addressing health disparities
Perkins et al.	2008	Assess patients research priorities for palliative care	East Anglia (United Kingdom of Great Britain and Northern Ireland)	Patients (*n* = 112)	Questionnaire	Statistical Analysis	Questions 1. Emergency: 2. Pain Control: 3. Helping doctors to hear and understand what patients are saying Thematic areas ranking: 1. Talking with patients 2. Medication 3. Symptoms 4. Help for patients/ families
Pillemer et al.	2015	To identify knowledge gaps and types of studies that should be conducted to improve providers’ ability to deliver palliative care most effectively.	New York, United States of America	Researchers (*n* = 18) Practitioners (*n* = 65)	Research-Practice Consensus Workshop	Ranking and consensus	• Research to improve individual-level palliative care practice • Research is needed on the physiology of the end of life, including nutrition, hydration, and oxygen, and on nonpharmacological approaches, including complementary and alternative therapies. • Research to improve system-level palliative care practice and capacity • Research on societal context for palliative care in the United States
Powell et al.	2014	To develop a prioritized research agenda for palliative care in Africa.	Africa	Palliative care professionals and Researchers Phase 1: (*n* = 49) Phase 2: (*n* = 14)	Phase 1: Consultative workshop Phase 2: Prioritization using a consensus development process.	Descriptive analysis	Three broad thematic areas were identified: • Patient, family, and volunteers • Health providers • Health systems
Riffin et al.	2015	To identify important directions for future research and inform the development of effective health policy and clinical practice in palliative care.	International Literature	n/a	Innovative Analytic Approach (Systematic Review technique)	Immersion--- crystallization framework	The identified research recommendations fell into 2 distinct, broad themes: • ways in which research methodological approaches should be improved • specific topic areas in need of future study
Shipman et al.	2008	To investigate what was understood by generalist end of life care and the current concerns and preferences for service research and development from the perspectives of clinicians, user groups, commissioners, academics and policy makers.	United Kingdom – London, East of England, Warwickshire and Scotland	210 participants including: health and social care practitioners; service commissioners; policy makers; academics; user and voluntary groups	National consultation and prioritisation exercise using a modified form of nominal group technique. Semi-structured questionnaires administered by email and telephone/face = to-face interviews	Thematic analysis	Research priorities identified in generalist end of life care included: • The need to improve service provision, including out of hours care • Identification of a model of care to address the supportive and palliative care needs of non-cancer patients in the community • Place of care and death and the associated costs and resources to be supported within national policies on care delivery • Understanding of patients and carers’ experiences
Sullivan et al.	2018	To gain a consensus on the research priorities of palliative care clinicians and researchers with a view to establish a prioritised research agenda for adult palliative care in Australia	Australia	25, 14 and 13 panelists (experts in palliative care research and/or practice in Australia) in rounds 1, 2 and 3 respectively.	A modified three round Delphi survey using questionnaires administered online	Statistical analysis.	Research priorities which emerged from the three rounds were ranked in order to priority to the top ten listed as: • To develop communication which facilitates patients’ and families’ understanding of transition from active treatment to palliative care • To improve the communication of accurate information about prognosis to patients when diagnosed • To improve palliative care for indigenous communities • To establish palliative care models for those who wish to remain at home but have significant care needs outside of care provided routinely • routine and formal identification and addressing of family caregivers’ support needs during the palliative care trajectory • to investigate how the aged care sector can identify and provide for the potentially chronic end-of-life support to aged people with multiple comorbid conditions but without a clear diagnosis for palliative intervention • to improve patients’ and families’ involvement in decisions regarding care in the last week of life • to explore cross-cultural approaches to terminal illness, death and dying and how these can inform palliative care • to assess the impact of the legislation on assisted dying on family decision-making and bereavement outcomes • to improve bereavement care in rural, remote and aboriginal populations

^**a**^More detailed descriptions of priorities were included in the thematic synthesis

## Abbreviations

CINAHL: Cumulative Index of Nursing and Allied Health Literature
EMBASE: Excerpta Medica database
HCP: Health Care Professionals
JBI: Joanna Briggs Institute
LMIC: Lower and Middle-Income Countries
OOH: Out of Hours (OOH)
PRISMA: *Preferred Reporting Items* for Systematic Reviews and Meta-Analyses
U.S.A: United States of America
UK: United Kingdom
